# Evaluation of Individual T1w-DIXON Contrasts for Subtraction Generation in Dynamic Contrast-Enhanced Breast MRI

**DOI:** 10.3390/diagnostics16081145

**Published:** 2026-04-12

**Authors:** Shirley-Maria Christian, Sebastian Bickelhaupt, Dominique Hadler, Lorenz A. Kapsner, Michael Uder, Frederik B. Laun, Sabine Ohlmeyer

**Affiliations:** 1 Gynecology Department, Klinikum Darmstadt, 64283 Darmstadt, Germany; 2 Institute of Radiology, Universitätsklinikum Erlangen, Friedrich-Alexander-Universität Erlangen-Nürnberg (FAU), 91054 Erlangen, Germany; sebastian.bickelhaupt@uk-erlangen.de (S.B.); dominique.hadler@uk-erlangen.de (D.H.); lorenz.kapsner@uk-erlangen.de (L.A.K.); michael.uder@uk-erlangen.de (M.U.); frederik.laun@uk-erlangen.de (F.B.L.); sabine.ohlmeyer@uk-erlangen.de (S.O.); 3 Medical Informatics, Friedrich-Alexander-Universität Erlangen-Nürnberg (FAU), 91054 Erlangen, Germany

**Keywords:** maximum intensity projection, DIXON, magnetic resonance imaging, only water

## Abstract

**Background/Objectives:** To evaluate the influence of different DIXON contrasts on the quality of subtraction images in dynamic breast MRI using maximum intensity projections (MIPs). **Methods:** This retrospective study included n = 40 women (median age: 53.5 years, range 23–83) undergoing clinically indicated breast MRI (3T). For each MRI examination, two independent readers individually evaluated GBCA-enhanced subtraction MIPS for different timepoints (n = 5) and DIXON contrasts (n = 4) per breast, resulting in a total of 800 individual evaluations. Evaluations comprised (a) qualitative measures, using Likert-scores for artefact strength, breast parenchyma visibility, lesion visibility and reading confidence; and (b) signal intensity, measured in three regions of interest with the apparent signal-to-noise ratio (aSNR) and apparent contrast-to-noise ratio (aCNR) calculated. The evaluation results were analysed to identify differences between DIXON contrasts. **Results:** The “only water” DIXON contrast at ~120s after GBCA injection achieved the highest lesion conspicuity and reading confidence scores and lowest artefact scores; however, its performance was not statistically significant (*p* > 0.05) compared to the “in-phase” and “opposed-phase” subtractions. The aCNR at the second timepoint was slightly, but not significantly (*p* > 0.05), lower than the first timepoint, whilst aSNR increased significantly from the first to second timepoint in all contrasts. **Conclusions:** Subtraction MIPs derived from the “only water” DIXON contrast achieved the highest qualitative scoring for lesion conspicuity and confidence, with the aSNR increasing and aCNR decreasing between the first and second timepoints.

## 1. Introduction

Breast cancer is the most common cancer among women [1]. Screening programs using X-ray mammography have been introduced in many countries; however, this method can be limited in women with high breast density [2,3]. Other imaging modalities are therefore increasingly being investigated for their complementary value in moving screening towards personalised care, including digital breast tomosynthesis, breast ultrasonography, and diagnostic breast MRI. Studies suggest that breast MRI provides increased sensitivity in cancer detection, especially for smaller tumours at earlier stages and in women with extremely dense breast tissue [4,5]. However, there is a reported risk of false positives, high cost, and long examination and interpretation times for routine multiparametric breast MRI protocols [5,6].

There has therefore been increasing interest in investigating contrast-enhanced (CE) abbreviated breast MRI protocols. Initial screening in such approaches is facilitated by using so-called maximum intensity projections (MIPs), derived from gadolinium-based contrast agent (GBCA)-enhanced T1-weighted subtractions. A significant reduction in reading time has been suggested with this approach, while also preserving high diagnostic performance and confidence [7,8]. Abbreviated protocols analysing MIPs are therefore considered a potential diagnostic approach for implementing breast MRI in more personalised screening settings [4,9].

T1-weighted GBCA-enhanced breast MRI can include the use of DIXON acquisitions. The DIXON acquisition relies on the chemical shift difference between water and fat and can be used to generate images by selectively suppressing water or fat signal [10,11]. A single DIXON acquisition allows one to reconstruct four individual image contrasts, reflecting different tissue properties: “in-phase”, “opposed-phase”, “fat-only”, and “water-only”. Acquiring dynamic CE breast MRI with the DIXON technique therefore provides various subtraction combinations reflecting different characteristics [12,13]. This raises the question of whether subtraction data—and subsequent diagnostic evaluation—are significantly influenced by the choice of the DIXON contrast used to create the subtraction series. Recent studies highlight the fact that while DIXON imaging improves fat suppression and overall image quality in breast MRI, there is currently no standardised approach for selecting a DIXON contrast to generate MIPs for abbreviated protocols. This lack of standardisation may influence image quality, lesion conspicuity, and diagnostic confidence, especially as artefacts may accumulate during MIP generation, depending on the DIXON contrast chosen [4,14].

Therefore, our study aimed to evaluate differences between DIXON-derived contrasts used for CE T1-weighted subtractions in breast MRI. We qualitatively and quantitatively investigated how these contrasts influence image quality, lesion visibility, and reader confidence. In addition, we assessed the dependence of these outcomes on the timing of acquisition after GBCA administration using a multi-reader study.

## 2. Materials and Methods

### 2.1. Study Sample and Ethics Approval

This institutional review board (IRB)-approved retrospective study included 40 women undergoing clinically indicated breast MRI at a large tertiary university hospital. The inclusion criteria were: 1. the patient received a breast MRI scan between October 2015 and December 2019; 2. a T1-weighted acquisition with DIXON contrasts was used for the dynamic contrast-enhanced (DCE) series; 3. the patient presented with a lesion corresponding to a BI-RADS score of 3 or higher in the clinical report by the radiologists, suitable for evaluation. For each patient, MIPs from all acquired timepoints of the DCE series after GBCA injection, and for all four individual DIXON contrasts (“in-phase”, “opposed-phase”, “water-only” and “fat-only”), were evaluated qualitatively and quantitatively, resulting in 20 MIPs evaluated per patient and 800 MIP images in the cohort. The study cohort has been previously reported on in a breast MRI study; however, DIXON contrasts in subtraction MIPs were not compared [blinded].

### 2.2. MRI Protocol

Clinically indicated breast MRI examinations were performed using a 3T MRI scanner (Magnetom Vida, Siemens Healthineers, Erlangen, Germany) in the prone position, using a clinical routine 18CH breast coil (Siemens Healthineers, Erlangen, Germany). The routine multiparametric protocol included the following sequences: unenhanced T2-weighted acquisition with fat saturation, unenhanced T1-weighted DIXON sequence—performed once before and five times after intravenous injection of GBCA (Gadovist, 0.1 mmol/kg body weight, flow rate 2.0 mL/s)—and diffusion weighted imaging (DWI) with three b-values (50, 750, and 1500 s/mm^2^). Timepoints were approximately 60 s apart, at 60 s, 120 s, 180 s, 240 s, and 300 s after GBCA injection. The full acquisition protocol is given in Table 1.

### 2.3. Data Analysis

For each T1w-DIXON post-contrast image, subtraction images were computed using the scanner software. The breast MRI data were then transferred to a research workstation running a Picture Archiving and Communication System (PACS) for further analysis. For each of the T1w-DIXON contrast-derived subtraction volumes, an MIP was individually generated in the direction of the slice axis. This resulted in a 2D representation of voxels with the highest intensities along the *z*-axis (i.e., head-to-feet).

Data analysis was performed independently by two readers, who were blinded to both the contrast type and the acquisition timepoint and had no access to the individual slice acquisitions, clinical information, or complementary data (R1: board-certified radiologist with >5 years of experience; R2: medical doctoral student with 1 year of experience in breast MRI data analysis). Each MIP was assessed independently using predefined Likert scales.

The MIPs of the T1-weighted GBCA-enhanced DIXON subtractions were analysed using four different subtraction DIXON contrasts: “in-phase”, “opposed-phase”, “only fat”, and “only water”. Accordingly, 20 subtraction MIPs were evaluated per patient (n = 40), resulting in 800 datapoints.

#### 2.3.1. Qualitative Reading

For the qualitative analysis, the readers individually and independently used Likert-scales to assess the following four scores: artefact strength in the respective MIPs derived from each DIXON contrast, lesion conspicuity/visibility, reading confidence, and background parenchymal enhancement (BPE). Artefact strength, lesion conspicuity/visibility, and reader confidence were evaluated using a 5-point Likert-scale after providing a joint primer to both readers to determine joint characteristics or individual scoring values. BPE was evaluated according to standard BI-RADS characteristics using the ACR BI-RADS Atlas [15,16]. Details on each score are given in Table 2.

#### 2.3.2. Quantitative Assessment

Quantitative analysis was performed by R2 under the supervision of R1. Three regions of interest (ROIs) were placed carefully in each subtraction MIP using the manual segmentation function of the open-source software, Slicer 3D 4.10 (www.slicer.org), following a standardised placement strategy to ensure reproducibility. One ROI was placed within the target lesion, adapted individually depending on lesion size, covering its entire volume while avoiding margins crossing visible lesion borders. An additional ROI was placed in the fibroglandular parenchyma of the contralateral, lesion-free breast. The ROI diameter was set to 2 cm, chosen to fit appropriately in all breasts regardless of their size and applied consistently across all patients. For the noise estimation, one circular ROI with a standardised diameter of 5 cm was placed in the background outside of the breast, centred along the midline of the sternum to ensure consistent positioning.

Readers were not allowed to modify window or visualisation settings, as this approach was intentionally chosen to reflect the conditions of an abbreviated MRI protocol, in which reliable assessment should be feasible without additional image manipulation. Given the exploratory study design and the limited sample size, no formal correction for multiple comparisons was applied, and the results were interpreted accordingly.

From these measurements, parameters of apparent signal-to-noise ratio (aSNR) and apparent contrast-to-noise ratio (aCNR) were derived [17,18]:

aSNR, measuring lesion signal against background noise, was calculated as follows:

$$aSNR=\frac{mean\;signal\;in\;lesion-mean\;noise\;signal}{standard\;deviation\;of\;noise\;signal}.$$

aCNR, measuring lesion signal against benign tissue, was calculated as follows:

$$aCNR=\frac{mean\;signal\;in\;lesion-mean\;signal\;normal\;tissue}{standard\;deviation\;of\;signal\;in\;normal\;tissue}.$$

### 2.4. Statistical Analysis

Statistics were calculated using SigmaPlot (V.14, Systat Software). Descriptive statistics are presented as either median with range or mean with standard deviation (SD) in parentheses. Statistical comparisons were performed after normality testing using the Shapiro–Wilk test. For visual reading scores, the Kruskal–Wallis one-way analysis of variance by ranks was used to assess differences between timepoints and contrasts. The apparent lesion to healthy FGT ratio (aCNR) was analysed using one-way repeated measures analysis of variance, after normality assumptions were violated according to the Shapiro–Wilk test (*p* < 0.05). Tukey’s test was used for individual pairwise comparisons. Significance level was set as *p* < 0.05.

## 3. Results

### 3.1. Study Population

Among the MRI examinations of 40 women (mean age: 51.58, SD ± 13.36 years), six presented with a BI-RADS of three, 14 presented with a BI-RADS of four, nine with a BI-RADS of five, and 11 with known malignancy at the time of examination (BI-RADS of six). Among the BI-RADS 4 lesions, nine were histopathologically confirmed to be malignant, and five were benign. Of the BI-RADS 5 lesions, seven were determined to be malignant and two benign by histopathology. All BI-RADS 3 lesions were confirmed as benign in the follow-up examinations. Thus, among our study population, 27 cases were found to be malignant, and 13 were benign. Among the findings, 32 were mass enhancements, and eight were non-mass enhancements. The mean size was 23 mm (±18 mm) for malignant and 19 mm (±15 mm) for benign lesions. Most target lesions were located in the right breast (n = 22, 55%), whilst 45% (n = 18) were located in the left. Further details are given in Table 3.

### 3.2. Qualitative and Quantitative Assessments of DIXON Subtraction Data

#### 3.2.1. Lesion Conspicuity and Reading Confidence in MIPs Derived from the Different DIXON Contrasts and Timepoints

For all DIXON contrasts, the highest lesion conspicuity was attributed by both readers to the second timepoint (approximately 120 s after GBCA injection; Table 4). When comparing the conspicuity scores between the different DIXON contrasts, the highest average score was given by both readers in the “water-only” (R1 4.50 (SD = 0.68), R2 4.46 (SD = 0.75)) and “opposed-phase” (R1 4.5 (SD = 0.98), R2 4.3 (SD = 0.83)) derived MIPs at the second timepoint; there was no statistically significant difference between the two contrasts (*p* > 0.05). The lowest lesion conspicuity was generally described by both readers for the latest (fifth) timepoint for all DIXON contrasts.

The confidence scores similarly indicated the highest confidence for “water-only” and “opposed-phase” subtraction MIPs at the second acquisition timepoint. The overall highest confidence scores were observed for the “water-only” contrast (Table 5).

#### 3.2.2. Artefacts in MIPs Derived from the Different DIXON Contrasts and Timepoints

**Figure 1:**
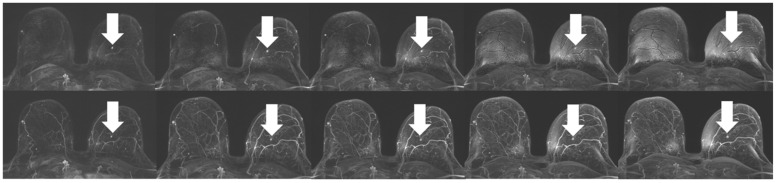
Example of a woman with a BI-RADS 3 lesion (mastopathy) in the left breast, displayed with the two best performing DIXON subtraction contrasts, demonstrating the identified characteristics: the upper row shows the “opposed-phase” subtraction MIPs over time, generally demonstrating reduced depiction of blood vessels; however, compared to the “water-only” contrast subtraction MIPs, “opposed-phase” exhibits a higher level of artefacts.

**Figure 2:**
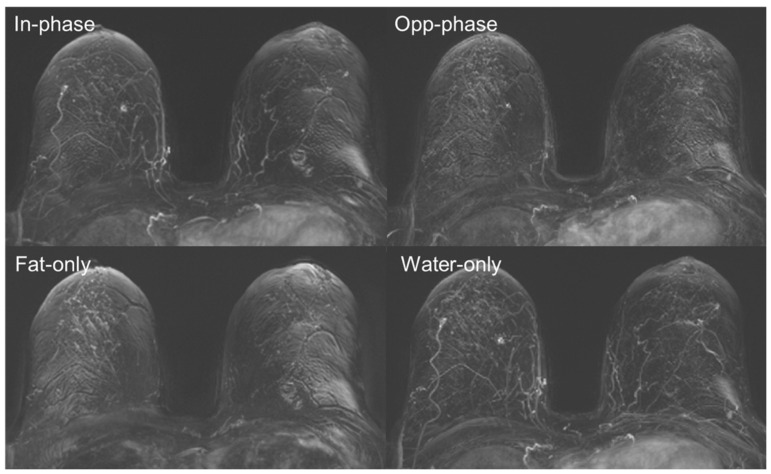
Example of a woman with small foci in the right breast. This demonstrates the different visibility of small enhancing targets in respective DIXON subtraction contrasts at the first timepoint after GBCA administration, with the lowest visibility apparent in the “fat-only” subtraction images.

Analysis of artefacts in the different DIXON subtraction MIPs revealed significant differences between the groups for both readers (each *p* < 0.05), with the “water-only” subtractions providing the lowest artefact scores for both readers and all DIXON contrasts. A tendency was shown towards increasing artefact strength with increasing examination time and thus subsequently acquired timepoints (Figure 1 and Figure 2). Full details for the results of both readers are given in Table 6.

#### 3.2.3. Background Parenchymal Enhancement (BPE) Scoring in the MIPs

BPE increased over time, though without statistically significant differences (*p* > 0.05) between DIXON contrasts. All DIXON contrasts demonstrated the highest levels of enhancement in the later phases (timepoint 4 or 5 after GBCA injection). However, a slight non-significant tendency for elevated BPE scores was observed for “water-only” subtraction data compared to other contrasts (details given in Table 7).

**Figure 3:**
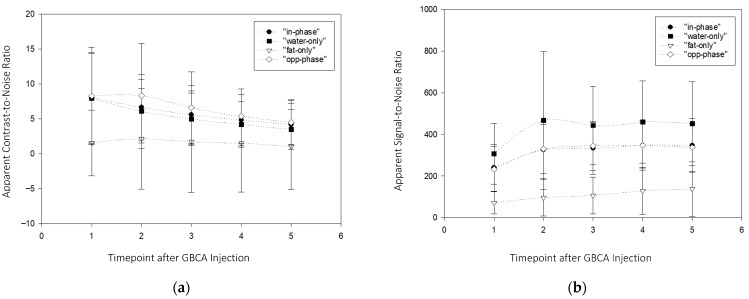
(**a**) The change in the apparent (measured) contrast-to-noise ratio for different timepoints in respective DIXON contrasts after GBCA injection. Boxes indicate mean, with the whiskers indicating the standard deviation of the respective timepoint and DIXON contrast, as shown in the upper right box. (**b**) The change in the apparent (measured) signal-to-noise ratio over different timepoints in the respective DIXON contrasts after GBCA injection. Boxes indicate mean, with the whiskers indicating the standard deviation of the respective timepoint and DIXON contrast, as shown in the upper right box.

Semiquantitative results revealed that the aCNR measured for all DIXON contrasts was highest in the first timepoint after contrast agent administration, followed by the second (Figure 3a). One-way repeated measure ANOVA indicated statistically significant differences within the entire dataset; however, within individual DIXON contrasts, no significant differences were observed between first and second timepoints, nor for comparisons between “water-only”, “in-phase”, and “opposed-phase” contrasts for the first and second timepoints (*p*-value for pairwise intra-contrast and inter-contrast comparison, each *p* > 0.05). The overall highest aSNR was observed for the “water-only” subtraction data; however, the peak aSNR at the second timepoint was not statistically significant from the “in-phase” and “opposed-phase” aSNR (*p* > 0.08 and 0.15) (Figure 3b). Full details are given in Table 8 and Table 9.

A summary of the key results is given in Table 10, indicating that target lesion visibility, reading confidence, artefact scores, and artefact area were all scored highest at Timepoint 2 (about 120 s after GBCA injection) in the “water-only” subtraction DIXON images.

## 4. Discussion

Using CE breast MRI after GBCA injection allows the generation of subtraction images that facilitate the detection and characterisation of suspicious findings. Increasingly, subtraction data are merged to form MIPs, speeding up the assessment process, as shown in studies investigating abbreviated breast MRI as a screening protocol [4].

In this retrospective study, we investigated whether the selection of subtraction data displayed as MIPs from different DIXON contrasts influenced the qualitative and quantitative assessment of suspicious lesions. In our multi-reader study, lesion conspicuity/visibility was rated highest in Timepoint 2 (reflective of about 120 s after GBCA injection) for all subtraction contrasts. When comparing different DIXON subtractions at this timepoint, the “water-only” and “opposed-phase” derived subtraction MIPs received numerically higher scores compared to other contrasts. While these differences were not statistically significant (*p* > 0,05), the results indicated a consistent trend favouring the “water-only” contrast. Meanwhile, reading confidence was also rated highest at this timepoint, with the “water-only” subtraction MIPs achieving the highest confidence scores, reinforcing their potential clinical relevance. In contrast, the apparent lesion-to-healthy FGT tissue ratio measurements indicated a slightly higher ratio for “opposed-phase” subtraction MIPs, although these differences were also not statistically significant. This limits the possible conclusions from these results, but does not contradict the observed preference for “water-only” in qualitative assessments. Artefact reading demonstrated an increase in those visible with increasing time after GBCA injection, with the “water-only” and “opposed-phase” subtractions achieving the lowest scores; this likely reflects the benefit of fat suppression in “water-only” images. This was accommodated by an increase in BPE over time for all contrasts.

CE subtraction data allow for visualisation of only the enhanced component of the breast tissue, and thus support visual lesion detection, as suspicious lesions are commonly characterised by altered biological tissue perfusion. Subtraction data can further be processed to derive MIPs for lesion detection [13]. A pivotal study by Kuhl et al. demonstrated that MIPs allow radiologists to rapidly determine the presence of potentially relevant lesions that may require further evaluation [4]. This shortened evaluation time can significantly reduce clinical work processing time for radiologists and associated costs. As a result, MRI could be used more frequently for screening and control examinations, particularly due to its high visual sensitivity for lesions from 4 mm in diameter, compared to other methods such as mammography and ultrasound [19,20,21,22].

However, breast MRI examinations are acquired in a dynamic manner and with varying acquisition techniques, posing challenges regarding optimal lesion detection. In their review, Mann et al. describe optimal detection timing as approximately 60–90 s after GBCA injection [13]. The DIXON method is one acquisition approach used to derive CE T1w subtraction images after GBCA injection [23,24]. It was developed as early as the 1980s and has several advantages, especially its uniform fat suppression and the generation of fat-saturated and fat-unsaturated images with only a single acquisition [25]. This reduces scanning time (compared to individually acquired sequences with and without fat suppression), and phase shifting allows a total of four contrasts to be identified. This could support diagnostic value in breast MRI by providing complementary tissue information [26,27,28].

DIXON contrasts can be processed into subtraction images in combination with dynamic acquisition spanning several minutes. It is therefore critical to identify optimal timepoints and contrasts for lesion detection. Our study demonstrated that, for subtraction contrasts, the second timepoint (approximately 120 s after GBCA injection) provided the highest qualitative lesion visibility/conspicuity and the highest reading confidence within all individual DIXON contrasts (jointly with the later timepoint 3 for the “in-phase” subtraction). Reading confidence and visibility were jointly rated highest overall for the “water-only” subtraction data, followed by the “opposed-phase” data. However, these findings should be interpreted as exploratory trends given the lack of statistical significance. Nevertheless, these results were also supported by the lowest artefact levels being recorded in the “water-only” and “opposed-phase” contrasts at the same timepoint. The lower artefact levels may be explained by the suppression of fat signal in the “water-only” contrast, which reduces high-intensity areas in cases of (subtle) co-registration errors, in contrast with “in-phase” images. In contrast, the semiquantitative measurements indicated a slight, but non-significant, increase in aCNR for the “opposed-phase” subtraction MIPs, and a decreasing trend from the first to the second timepoint, although this was not statistically significant. Overall, a tendency of increasing artefacts was observed over time; this may not be surprising given the relatively high prevalence of image artefacts in breast MRI and their accumulation in subtraction MIPs [14], which can be easily influenced by relatively subtle movements which lead to non-rigid deformation of breast shapes between acquisitions.

Our study has several limitations. First, the sample was relatively small (n = 40), limiting the statistical power and possibly contributing to the lack of significant differences between contrasts. However, the assessment and analysis were designed for a rather comprehensive vertical-depth approach, investigating a total of 800 datapoints and providing insight into detailed characteristics of different DIXON contrasts. Further, our study included only two readers, of whom only one was a board-certified radiologist. This was addressed by a preparatory meeting of the readers to establish joint criteria for the visual reading, and by including quantitative assessments to reduce the dependency on qualitative readings. Additional inter-reader agreement analysis using Cohen’s kappa indicated slight to fair agreement across the evaluated qualitative scores (κ ranging from 0.03 to 0.29), suggesting some degree of reader variability, which is not unexpected given the differing levels of reader experience and the subjective nature of Likert-based image assessment. However, despite this variability, both readers demonstrated consistent overall trends in the relative assessment of DIXON contrasts and optimal timepoints, supporting the robustness of the main findings. Furthermore, our study only included DIXON contrast data. Thus, we cannot assess whether other T1 acquisition techniques, such as spectral fat suppression, might provide similar results. As we only included a single type of MRI device, we also cannot draw conclusions on other devices or field strengths. Furthermore, no correction for multiple comparisons was applied, increasing the risk of type I error. Additionally, complete blinding to contrast type and timepoint was not feasible due to inherent image characteristics.

Our findings indicate that the second post-contrast timepoint—approximately 120 s after GBCA administration—may provide the highest visually rated lesion conspicuity and diagnostic confidence for detecting suspicious lesions on DIXON subtraction MIPs in breast MRI. Among the evaluated DIXON contrast reconstructions, “water-only” images consistently performed best in visual assessments, followed by “opposed-phase” images; despite the lack of statistical significance, these results clarify the main message of contrast preference. Semi-quantitative measures (aSNR and aCNR) did not differ significantly between contrasts, suggesting that visual image quality and reader confidence may not always correlate directly with signal-based metrics, highlighting the importance of contrast selection in clinical image interpretation. These findings further support the rationale for combining DIXON and GBCA in breast MRI, as their complementary use enhances lesion visualisation and reader confidence, particularly for preferred “water-only” subtraction MIPs at the 120 s post-contrast timepoint.

These results are particularly relevant in the context of abbreviated breast MRI protocols, which are being increasingly adopted to improve accessibility and reduce reading times, especially in high-volume or screening settings [29,30,31,32]. If confirmed in larger cohorts, identification of the most diagnostically valuable post-contrast timepoint and reconstruction technique may help refine abbreviated MRI protocols. By prioritising specific timepoints and contrast reconstructions, acquisition or interpretation time could potentially be reduced. Prior studies have shown that abbreviated protocols can maintain diagnostic accuracy while significantly reducing acquisition and interpretation time, making them well-suited for high-risk screening populations [32,33].

Moreover, the consistent performance of DIXON MIPs—particularly “water-only” and “opposed-phase” contrasts—at the 120 s timepoint may offer advantages for AI-assisted or automated detection frameworks, which often rely on high-quality, standardised image inputs [34]. These findings emphasise the practical relevance of the “water-only” contrast, and may support hypothesis generation for optimising abbreviated MRI protocols and integrating advanced visualisation or computer-aided diagnostic tools into routine breast MRI workflows. Future studies should further evaluate these findings in larger, multicentre cohorts, and assess the impact of optimised DIXON-based MIPs on reader performance, particularly in abbreviated or AI-assisted screening environments.

## Figures and Tables

**Table 1:** MRI acquisition protocol.

Scanner/Coil Details	
MRI field strength	3T
MRI device	Siemens Magnetom Vida, Siemens Healthineers, Germany, Erlangen
Coil	18 Channel Breast Coil, Siemens Healthineers, Germany Erlangen
**T1x sequence details**	
Scanning sequence	T1w GRE
MR acquisition type	3D
Slice thickness	1.5 mm
Repetition time (TR)	5.41 ms
Echo time (TE)	3.46 ms
Number of averages	1
Percent phase field of view	100
Acquisition matrix	448 × 358
Flip angle	8°
Contrast agent (GBCA)	Gadovist (Bayer, Germany), intravenous s injection, 0.1 mmol/kg body weight, flow rate 2.0 mL/s
Abbreviations: mm = millimetres, ms = milliseconds	

**Table 2:** Scoring systems used for the visual evaluation.

Artefact Strength (Likert-Scores 1–5)	
1	Barely
2	Mild
3	Has the potential to slightly impair visibility of a lesion
4	Has the potential to significantly impair visibility of a lesion
5	So severe that lesions are completely invisible behind the artefact
**Background Enhancement**	
1	Minimal (adapted to BI-RADS ACR category A)
2	Mild (adapted to BI-RADS ACR category B)
3	Moderate (adapted to BI-RADS ACR category C)
4	Marked (adapted to BI-RADS ACR category D)
**Lesion Conspicuity/Visibility (Likert-scores 1–5)**	
1	Very poor
2	Poor
3	Fair
4	Good
5	Excellent
**Reading Confidence (Likert-scores 1–5)**	
1	Not confident at all
2	Slightly confident
3	Somewhat confident
4	Fairly confident
5	Completely confident

**Table 3:** Demographics.

Variable	Sample
*N* patients	40
MIPs evaluated	n = 800 (40 (patients) × 4 (contrasts) × 5 (timepoints))
Mean age (years)	51.58 (sd: ±13.36, range 23–83)
Mean weight (kg)	72.28 (sd: ±15.16)
Mean size (cm)	167.6 (sd: ± 6.85)
Mean contrast agent (Gadovist) (mL)	7.25 (sd: ± 1.55)
*N* lesions left breast	18
*N* lesions right breast	22
*N* BI-RADS Score 3	6
*N* BI-RADS Score 4	14
*N* BI-RADS Score 5	9
*N* BI-RADS Score 6	11

**Table 4:** Lesion conspicuity/visibility scores; mean with standard deviation in parentheses.

	In Phase		Opposed Phase		Water Only		Fat Only	
Timepoint	R1	R2	R1	R2	R1	R2	R1	R2
1	4.8 (1.17)	3.71 (1.39)	4.13 (1.18)	3.69 (1.15)	4.28 (1.13)	4.41 (1.06)	1.67 (1.15)	**
2	4.26 * (1.05)	3.87 * (1.24)	4.5 * (0.98)	4.3 * (0.83)	4.50 * (0.68)	4.46 * (0.75)	2.33 * (1.52)	**
3	3.94 (1.16)	3.82 (1.14)	4.4 (0.96)	4.17 (1.03)	4.20 (0.83)	4.46 (0.76)	2.33 (2.30)	**
4	3.75 (1.11)	3.64 (1.11)	4.05 (1.16)	3.85 (1.18)	3.74 (1.25)	4.00 (1.14)	2.33 (2.30)	**
5	3.45 (1.21)	3.52 (1.15)	3.75 (1.34)	3.66 (1.24)	3.48 (1.35)	3.74 (1.25)	2.33 (2.30)	**

* No statistical difference (*p* > 0.05) was observed between the Timepoint 2 lesion visibility scores for the individual readers and contrasts. ** Reader 2 identified too few lesions to calculate a mean (n = 1).

**Table 5:** Reading confidence scores; mean and standard deviation in parentheses.

	In Phase		Opposed Phase		Water Only		Fat Only	
Timepoint	R1	R2	R1	R2	R1	R2	R1	R2
1	4.40 (1.09)	3.92 (1.44)	4.29 (1.17)	3.97 (1.13)	4.47 (1.0)	4.46 (1.02)	1.67 (0.57)	**
2	4.47 * (1.03)	4.15 * (1.33)	4.58 * (0.90)	4.45 * (0.84)	4.71 * (0.6)	4.82 * (0.45)	2.66 * (1.52)	**
3	4.30 (1.03)	4.25 (1.16)	4.59 (0.81)	4.32 (0.99)	4.48 (0.82)	4.66 (0.57)	2.33 (2.30)	**
4	4.16 (1.19)	3.97 (1.16)	4.34 (0.99)	3.10 (1.21)	4.10 (1.20)	4.30 (1.05)	2.33 (2.30)	**
5	3.94 (1.31)	3.88 (1.29)	4.10 (1.28)	4.00 (1.25)	3.92 (1.42)	4.02 (1.24)	2.33 (2.30)	**

* No statistical difference (*p* > 0.05) was observed between the Timepoint 2 lesion visibility scores for the individual readers and contrasts. ** Reader 2 identified too few lesions to calculate a mean (n = 1).

**Table 6:** Artefacts visible in the DIXON subtraction MIPs; mean with standard deviation in parentheses.

	In Phase		Opposed Phase		Water Only		Fat Only	
Timepoint	R1	R2	R1	R2	R1	R2	R1	R2
1	2.88 (1.45)	2.20 (1.15)	3.17 (1.13)	2.13 (1.02)	4.47 (1.0)	4.46 (1.02)	1.67 (0.57)	**
2	3.19 * (1.27)	2.66 * (1.10)	3.00 * (1.20)	2.20 * (1.00)	4.71 * (0.6)	4.82 * (0.45)	2.66 * (1.52)	**
3	3.38 (1.31)	2.70 (1.03)	3.22 (1.15)	2.35 (0.95)	4.48 (0.82)	4.66 (0.57)	2.33 (2.30)	**
4	3.60 (1.28)	1.96 (0.94)	3.25 (1.16)	2.38 (0.96)	4.10 (1.20)	4.30 (1.05)	2.33 (2.30)	**
5	3.69 (1.26)	3.20 (0.89)	3.28 (1.22)	2.63 (0.93)	3.92 (1.42)	4.02 (1.24)	2.33 (2.30)	**

* *p* < 0.001 as compared to the “fat-only” contrast at Timepoint 2. Increasing scores indicate increasing levels of artefacts. ** Reader 2 identified too few lesions to calculate a mean (n = 1).

**Table 7:** Background parenchymal enhancement in DIXON subtraction MIPs; mean with standard deviation in parentheses.

	In Phase		Opposed Phase		Water Only		Fat Only	
Timepoint	R1	R2	R1	R2	R1	R2	R1	R2
1	1.61 (0.91)	1.74 (0.82)	1.73 (1.02)	1.57 (0.87)	1.58 (0.95)	2.01 (0.87)	2.00 (0)	1.25 (0.50)
2	1.71 (0.98)	2.06 (0.82)	1.93 (1.01)	2.00 (1.02)	1.76 (0.98)	2.39 (0.98)	1.5 (0.70)	1.60 (0.54)
3	1.84 (1.03)	2.29 (0.94)	1.97 (1.02)	2.17 (1.05)	1.83 (0.99)	2.47 (0.99)	2.00 (0)	1.75 (0.50)
4	1.93 (1.03)	2.28 (0.95)	2.10 (1.14)	2.20 (1.06)	1.87 (1.03)	2.60 (1.00)	2.00 (0)	2.0 (0)
5	2.11 (1.10)	2.34 (0.97)	2.09 (1.13)	2.38 (1.03)	1.88 (1.05)	2.74 (0.95)	2.00 (0)	2.2 (0.44)

**Table 8:** Apparent lesion to healthy FGT signal ratio (aCNR)**;** s = seconds after injection of gadolinium-based contrast agent (GBCA); mean with standard deviation in parentheses.

		In Phase	Opposed Phase	Water Only	Fat Only
Timepoint	Time	Mean (SD)			
1	~60 s	8.05 (6.4) *	8.27 (6.95) *	7.93 (6.49) *	1.55 (4.7)
2	~120 s	6.65 (4.7) *	8.31 (7.55) *	6.09 (4.5) *	2.1 (7.2)
3	~180 s	5.57 (4.1)	6.61 (5.10)	4.96 (3.76)	1.72 (7.25)
4	~240 s	4.82 (3.70)	5.38 (3.89)	4.2 (3.26)	1.51 (6.99)
5	~300 s	4.20 (3.57)	4.49 (3.16)	3.47 (2.83)	1.02 (6.15)

* No significant difference (*p* > 0.05) was found between the “in-phase”, “water-only” and “opposed-phase” DIXON contrasts for Timepoints 1 and 2.

**Table 9:** Apparent signal-to-noise ratio (aSNR); s = seconds after injection of gadolinium-based contrast agent (GBCA). Mean with standard deviation in parentheses.

		In Phase	Opposed Phase	Water Only	Fat Only
Timepoint	Time	Mean (SD)			
1	~60 s	238.76 (112.1) **	233.11 (107.8) **	306.74 (145.23) **	70.87 (53.88)
2	~120 s	324.69 (134.33) *	329.23 (117.02) *	466.98 (331.46) *	96.24 (89.09) *
3	~180 s	333.42 (125.54)	344.31 (117.98)	442.76 (188.23)	106.45 (88.17)
4	~240 s	347.60 (119.0)	346.64 (109.15)	459.14 (197.29)	128.59 (113.51)
5	~300 s	346.15 (128.96)	337.00 (114.34)	451.90 (201.17)	136.99 (130.53)

* No significant difference (*p* > 0.05) was found between the “in-phase”, “water-only” and “opposed-phase” DIXON contrasts for Timepoint 1. ** statistically lower than Timepoint 2 (*p* = 0.005 [“water only”], *p* = 0.009 [“opposed phase”] and 0.027 [“in phase”]).

**Table 10:** Key results summary table.

Score	Result
Lesion conspicuity/visibility	Highest in Timepoint 2 for all contrasts; “water-only” and “opposed-phase” derived subtraction MIPs rated as highest regarding lesion conspicuity. Semiquantitative measurements indicate that the “opposed-phase” subtraction MIPs provide the highest apparent contrast-to-noise ratio (aCNR) in the first two timepoints, without significant differences compared to the “water-only” and “in-phase” DIXON contrasts.
Reading confidence	Highest in Timepoint 2 and the “water-only” contrast; decreased for all contrasts with increasing time after GBCA injection.
Artifact scores	Lowest in Timepoint 1 and 2 and for “water-only” contrast, with one reader also scoring low artefact scores in the “opposed-phase” derived subtraction MIPs.
aSNR/aCNR	Apparent signal-to-noise ratio (aSNR) increased from Timepoint 1 to Timepoint 2; in contrast, aCNR slightly decreased between the timepoints, without a statistically significant difference.
Background enhancement	Lowest in Timepoint 1, increased over time.

## Data Availability

The data presented in this study are available on request from the corresponding author due to data security restrictions.

## Abbreviations

The following abbreviations are used in this manuscript:

aCNR	Apparent contrast-to-noise ratio
ACR	American College of Radiology
aSNR	Apparent signal-to-noise ratio
BI-RADS	Breast Imaging Reporting and Data System
CH	Channel
FGT	Fibroglandular tissue
GBCA	Gadolinium-based contrast agent
LBR	Lesion-to-background ratio
MIP	Maximum intensity projection
MRI	Magnetic Resonance Imaging
N	Number
Opp	Opposed
ROI	Region of interest
SD	Standard deviation
T	Tesla
